# Novel in-house knowledge-based automated planning system for lung cancer treated with intensity-modulated radiotherapy

**DOI:** 10.1007/s00066-023-02126-1

**Published:** 2023-08-21

**Authors:** Yan Shao, Jindong Guo, Jiyong Wang, Ying Huang, Wutian Gan, Xiaoying Zhang, Ge Wu, Dong Sun, Yu Gu, Qingtao Gu, Ning Jeff Yue, Guanli Yang, Guotong Xie, Zhiyong Xu

**Affiliations:** 1 Shanghai Chest Hospital, School of Medicine, Shanghai Jiao Tong University, Shanghai, China; 2 Shanghai Pulse Medical Technology Inc., Shanghai, China; 3 School of Physics and Technology, University of Wuhan, Wuhan, China; 4 School of Information Science and Engineering, Xiamen University, Xiamen, China; 5 Ping An Healthcare Technology Co. Ltd., Shanghai, China; 6 School of Biomedical Engineering, Health Science Center, Shenzhen University, Shenzhen, China; 7 School of Engineering, Hong Kong University of Science and Technology, Hong Kong SAR, China; 8 School of Medicine and Biological Information Engineering, Northeastern University, Shenyang, China; 9 Department of Radiation Oncology, Rutgers Cancer Institute of New Jersey, Rutgers University, New Brunswick, NJ USA; 10 Radiotherapy Department, Shandong Second Provincial General Hospital, Shandong University, Jinan, China; 11 Ping An Health Cloud Company Limited, Shanghai, China; 12 Ping An International Smart City Technology Co., Ltd., Shanghai, China

**Keywords:** Deep learning, Dose prediction, Knowledge-based planning, Lung cancer, Image retrieval

## Abstract

**Purpose:**

The goal of this study was to propose a knowledge-based planning system which could automatically design plans for lung cancer patients treated with intensity-modulated radiotherapy (IMRT).

**Methods and materials:**

From May 2018 to June 2020, 612 IMRT treatment plans of lung cancer patients were retrospectively selected to construct a planning database. Knowledge-based planning (KBP) architecture named αDiar was proposed in this study. It consisted of two parts separated by a firewall. One was the in-hospital workstation, and the other was the search engine in the cloud. Based on our previous study, A‑Net in the in-hospital workstation was used to generate predicted virtual dose images. A search engine including a three-dimensional convolutional neural network (3D CNN) was constructed to derive the feature vectors of dose images. By comparing the similarity of the features between virtual dose images and the clinical dose images in the database, the most similar feature was found. The optimization parameters (OPs) of the treatment plan corresponding to the most similar feature were assigned to the new plan, and the design of a new treatment plan was automatically completed. After αDiar was developed, we performed two studies. The first retrospective study was conducted to validate whether this architecture was qualified for clinical practice and involved 96 patients. The second comparative study was performed to investigate whether αDiar could assist dosimetrists in improving the quality of planning for the patients. Two dosimetrists were involved and designed plans for only one trial with and without αDiar; 26 patients were involved in this study.

**Results:**

The first study showed that about 54% (52/96) of the automatically generated plans would achieve the dosimetric constraints of the Radiation Therapy Oncology Group (RTOG) and about 93% (89/96) of the automatically generated plans would achieve the dosimetric constraints of the National Comprehensive Cancer Network (NCCN). The second study showed that the quality of treatment planning designed by junior dosimetrists was improved with the help of αDiar.

**Conclusions:**

Our results showed that αDiar was an effective tool to improve planning quality. Over half of the patients’ plans could be designed automatically. For the remaining patients, although the automatically designed plans did not fully meet the clinical requirements, their quality was also better than that of manual plans.

## Introduction

Artificial intelligence (AI) has been applied in medical imaging-based diagnosis and prognosis and has shown significant advantages with regard to application [1–5]. Although recent work has demonstrated the effectiveness of AI in radiotherapy [6], e.g., AI segmentation of planning target volume (PTV) and organs at risk (OARs) [7–10] and the AI prediction of dose images [11, 12], its application is still limited.

In clinical practice, a radiotherapy treatment plan is generated by configuring prescription, optimization algorithm, dose calculation algorithm and grid resolution, settings and options of the optimizer and optimization parameters (OPs) including field geometry, number of fields, and optimization goals. Then, the planner iteratively modifies OPs until the plan meets the clinical constraints. This is a very time-consuming and laborious process. And to some extent, the selection of the OPs and the plan modification process are based on the planner’s experience [13]. Thus, the quality of radiotherapy treatment plans may vary between planners, and some patients may be treated with suboptimal plans [14, 15].

In order to minimize the variations of plan quality between planners and improve the plan quality, automatic treatment planning (ATP) methods [16–18] were developed. Some commercial ATPs are available, such as RapidPlan [19] and HyperArc [20] from Varian, auto-planning [21] from Philips, RayStation autoplanning modules, EZfluence [22] from Radformation, and Elements [23] from Brainlab. Some other noncommercial systems were also developed by researchers, such as iCycle [24, 25] which utilized an a priori approach with multicriteria optimization. However, all these commercial and noncommercial systems still require planners to select OPs as the input to generate a treatment plan. However, because the selection of OPs depends largely on the experience of planners, suboptimal plans may result. Further work is needed to mitigate the dependence on planners and to generate invariant treatment plans of high quality.

In order to solve the above problems, researchers proposed several solutions to improve the quality, uniformity and processing efficiency of planning, such as the use of a complicated objective function, the use of multiobjective optimization and the introduction of knowledge-based automated planning methods (KBAP) [26–29]. Zhang et al. [30] proposed a semi-automatic radiotherapy treatment planning process by combining the ideas of machine learning automated planning and multicriteria optimization (MCO). In their workflow, handcrafted features were introduced. KBAP is a two-step approach to realize the automatic design of radiotherapy treatment planning. Clinically acceptable doses were first predicted and then the predictions were converted into deliverable plans with an optimization algorithm [31]. Babier et al. [32] developed and evaluated a new inversed optimization model. In this model, the weight of the objective function estimated from the clinical dose–volume histogram (DVH) was used to generate the inversed plan. DVH is two-dimensional and cannot include spatial information. Therefore, automated planning based on three-dimensional (3D) dose distribution may be more advantageous.

In the present study, we introduce a novel method named αDiar. It can automatically design plans for lung cancer patients treated with intensity-modulated radiotherapy (IMRT) based on predicted dose images. First, the optimal 3D dose distribution of the new plan was predicted based on the A‑Net model [33] developed by our team. Then an image retrieval model was constructed with AI. The features of the predicted 3D dose distribution and the dose distribution of the clinical plan in the database, respectively, were extracted with this model. Finally, the similarities between the features of predicted and clinical dose distributions were compared. The feature in the database that was most similar to the feature of the predicted 3D dose distribution was found, and its corresponding OPs were assigned to the new plan. Then the automated design of the new plan was completed.

Two experiments were conducted to evaluate and investigate the clinical applicability of this architecture. First, αDiar was used to automatically generate treatment plans. The quality of these auto-generated plans was evaluated to determine their clinical feasibility. Second, a comparison between plans with and without the assistance of αDiar was performed. To verify the potential value of αDiar, dosimetric parameters group of clinical metrics were calculated and compared with the clinical acceptance criteria. Both of the experiments comprised a pilot step to employ the technology of image retrieval in the automatic design of radiotherapy treatment planning, and the proposed architecture may enable automated treatment planning that does not rely on the experience of planners.

## Methods

### Database

From May 2018 to June 2020, 612 IMRT treatment plans of lung cancer patients were retrospectively selected. All these clinical plans were designed by three experienced dosimetrists. Each of these original clinical plans consisted of four to seven coplanar 6 MV photon beams. An Edge linear accelerator (Varian, Palo Alto, CA) was selected for dose delivery. All plans were normalized; thus, 95% of the PTV received 100% of the prescription dose. Each treatment plan consisted of a computed tomography (CT) scan, PTV contour(s), OAR contours, prescription dose, beam arrangement, optimization goals, and clinically delivered dose distribution that was calculated in the Pinnacle 9.10 treatment planning system (TPS; Philips Healthcare, Fitchburg, WI, USA). All the contours of PTV and OARs were delineated by junior radiation oncologists and reviewed by experienced radiation oncologists. OARs included left lung, right lung, total lung, spinal cord, and heart in this study. Total lung was defined as the union of left lung and right lung excluding gross tumor volume (GTV).

### Architecture

**Fig. 1:**
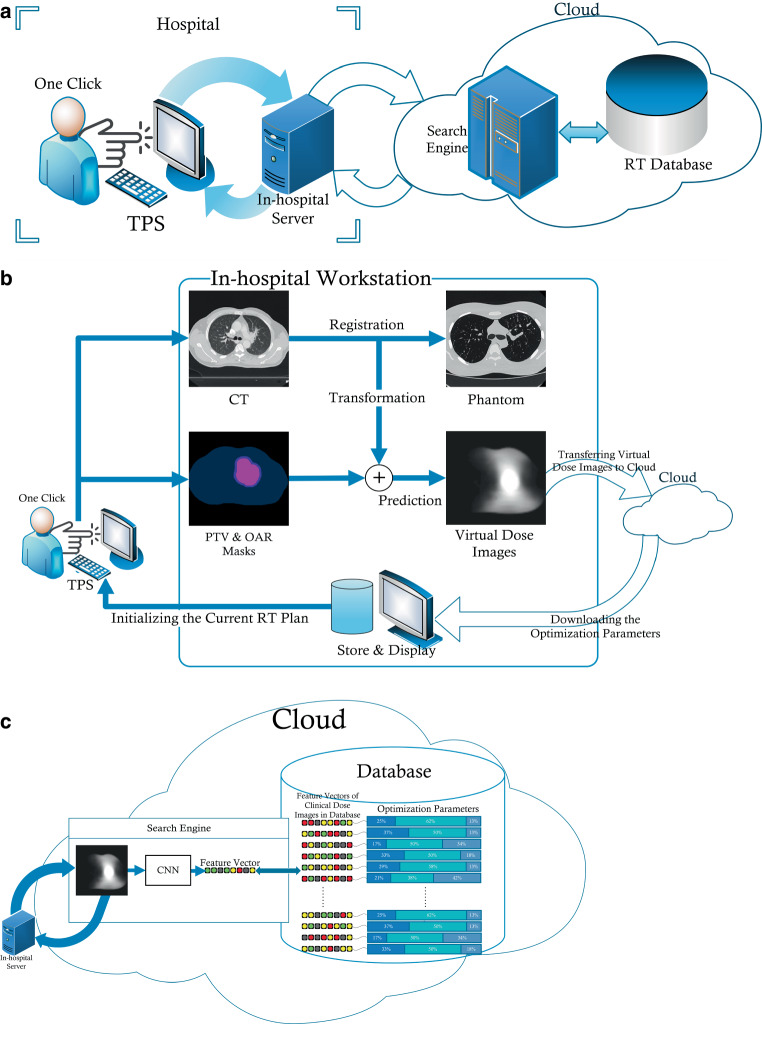
Architecture of dose–image agent retrieval. **a** αDiar’s workflow; **b** the workflow of in-hospital server; **c** the workflow in cloud

Figure 1 shows the architecture of αDiar, which is the automated knowledge-based treatment plan design system proposed in this study. The system contained two parts: the in-hospital workstation and the search engine in the cloud. In the in-hospital workstation, CT scan, PTV, and OARs contours of a new patient were transferred to the in-hospital workstation with only one click using a TPS script. The CT scan was registered with the phantom chest CT scan [34] via the registration toolbox—elastiX [35]. The transformation matrix gained from the registration was applied to the corresponding PTV and OARs masks afterward. The transformed PTV and OAR masks automatically predicted a series of virtual dose images through the dose prediction The AI model [33] was supervised by the ground truth of clinical dose images. With no other information, the predicted virtual dose images were subsequently transferred to the cloud for computation of a feature vector. To predict the feature vector, a 3D convolutional neural network (3D CNN) model was trained through the virtual and clinically delivered dose images. The derived feature vector of the virtual dose images was compared to the feature vectors derived from clinical dose images of treatment plans in the database. The Euclidean distances between the feature vectors of the virtual dose images and stored clinical dose images were calculated. A link between the feature vector of the virtual dose images and the most similar feature vectors (with the smallest Euclidean distance) in the database was established. The corresponding OPs of the most similar stored feature vectors were transferred back to the TPS through the in-hospital workstation. Finally, in the TPS, the auto-planning module was utilized to optimize the plan of the new patient with the downloaded OPs.

**Fig. 2:**
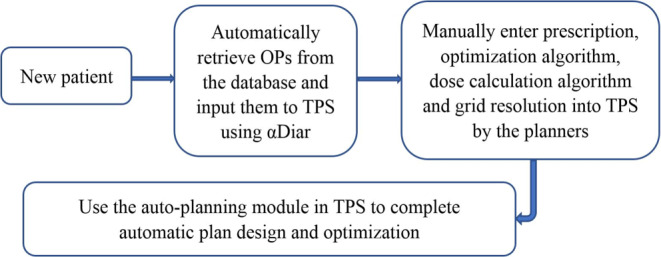
Flowchart of steps using αDiar

No human intervention was required in the design process of the radiotherapy plan. The optimal dose distribution prediction, OPs retrieval and plan optimization of the new plans were done entirely by the system and did not depend on the experience of planners. Thus, αDiar achieved a fully automatic design of treatment plans. The specific step-by-step flowchart is shown in Fig. 2.

### Prediction of virtual dose images

In previous research, A‑Net was used to predict virtual dose images from the contours of PTV and OARs [31] and included an encoding part and a decoding part in A‑Net. In the encoding part, there were four stages with two squeeze-and-excitation (SE) [36] blocks in each stage. And there were two stages in the decoding part. A‑Net is an end-to-end network through which the virtual images of a case can be predicted in only one try. Its inputs were the masks of PTV and OARs and the size of the input images was 384 × 384 × 128. Its outputs were the virtual dose images and the size of the output dose images was 96 × 96 × 128. During the process of model training, the ground truth was the clinically delivered dose images. The performance of A‑Net was demonstrated in the article [31].

### Building and training of image retrieval model

**Fig. 3:**
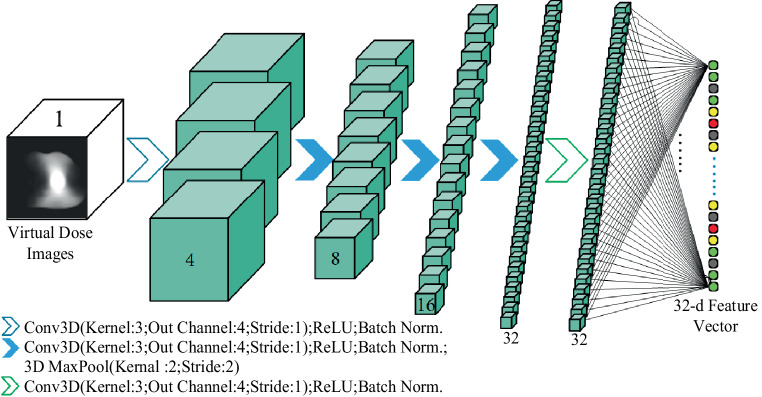
The three-dimensional convolutional neural network (3D CNN) used in image retrieval model

A small and simple 3D CNN was built in the training of the image retrieval model. The inputs were the dose images, and the output was the feature vector of the dose images. As shown in Fig. 3, there were five sets of convolutional layers. The size of the input images was 96 × 96 × 128. First, it went through a set of 3D convolution (kernel size: 3 × 3 × 3, the number of output channels: four times the number of input channels, stride: 1 × 1 × 1), ReLU, and a batch normalization (BN). Then it went through three blocks of 3D convolution and 3D max-pooling. Each 3D convolution (kernel size: 3 × 3 × 3, the number of output channels: doubling the input channels, stride: 1 × 1 × 1) was followed by a BN, and 3D max-pooling (kernel size: 2 × 2 × 2, stride: 2 × 2 × 2). Finally, a fully connected layer converted the feature map to a 32‑d feature vector, and the output was a feature vector with a size of 32 × 1.

In the training phase, the network consisted of three inputs: (i) anchors—the virtual dose images, denoted as *A*; (ii) positives—the clinically delivered dose images of the same patient to the virtual dose images, denoted as *P*; (iii) negatives—the clinically delivered dose images of another dissimilar patient, denoted as *N. A, P* and *N* composed a triplet [37]. *A, P* and *N* separately went through the image retrieval model and their corresponding feature vectors *a, p* and *n* were generated respectively. During the training phase, *A* and *P* were the virtual dose images and the clinically delivered dose images of the same patient. In case of the random selection of *N* may not train a robust AI model well, an online hard example mining procedure proposed in [38] was used in the selection of *N*. In a mini-batch, *N* could be selected from other triplets’ *A* and *P*.

The distance matrix in a mini-batch was defined by the following equation:

1 $$\left(\begin{array}{ccc} d\left(a_{1}{,}p_{1}\right) & \cdots & d\left(a_{1}{,}p_{k}\right)\\ \vdots & \ddots & \vdots \\ d\left(a_{k}{,}p_{1}\right) & \cdots & d\left(a_{k}{,}p_{k}\right) \end{array}\right)$$

where *k* was the batch size, *a*_*i*_ and *p*_*i*_ were the feature vectors of *i*th anchor and *i*th positive in the mini-batch, and *d*(*a*_*i*_,*p*_*j*_) was the distance between *a*_*i*_ and *p*_*j*_. *d*(*a*_*i*_,*p*_*j*_) was defined as:

2 $$d\left(a_{i}{,}p_{j}\right)=\sqrt{2-2a_{i}{p}_{j}^{T}}$$

In order to find a hard negative sample, the minimum distance amount *d*(*a*_*i*_,*p*_*j*_) and *d*(*a*_*q*_,*p*_*i*_) ($j{,}q=1\ldots k$) were denoted as *d*(*a*_*i*_,*p*_*jmin*_) and *d*(*a*_*qmin*_,*p*_*i*_). The triplet loss function could be written as:

3 $$Loss=\frac{1}{k}{\sum }_{i=1{,}n}^{n}\max\Big(0{,}\textit{margin}+d\left(a_{i}{,}p_{i}\right)\\ -\min\left(d\left(a_{i}{,}p_{\mathrm{jmin}}\right){,}d\left(a_{\mathrm{qmin}}{,}p_{i}\right)\right)\Big)$$

where the *margin* was a constant, set as 1.

The complete proposed network was trained on a Tesla V100 GPU with 16 GB memory and was randomly initialized with Glorot normal distribution. The Adam optimizer with “poly” learning rate decay policy was employed to minimize the loss function. The “poly” learning rate decay policy can be formulated as follows:

4 $$lr_{\text{itertion}}=lr_{\mathrm{init}}*\left(1+\frac{1}{\textit{decay*iteretion}}\right)$$

where *lr*_*itertion*_ was the learning rate in this iteration, *lr*_*init*_ was the initial learning rate assigned to 0.001, and *decay* was initialized to 0.01. Given the limitation of GPU memory size, the batch size was one.

### Inference of image retrieval model

In the inference phase, the trained image retrieval model was applied to infer the virtual dose images and the obtained feature vector was denoted as *f*_*v*_. The clinical dose images of each patient in the database were fed to this image retrieval model procedure and the output feature vectors were denoted as *f*_*c*_. When retrieving a plan, the Euclidean distance between *f*_*v*_ and every *f*_*c*_ in the database was calculated. The smaller the Euclidean distance was, the more similar the two features were. Finally, the OPs corresponding to the *f*_*c*_ that were most similar to *f*_*v*_ were used to generate planning for the new patient. The formula of Euclidean distance was as follows:

5 $$dist\left(X{,}Y\right)=\sqrt{{\sum }_{1}^{n}({x_{i}}-{y_{i)}}^{2}}$$

where X and Y represented the feature vector of the predicted virtual dose image and clinical dose images stored in the database, respectively.

### Study of fully automated usage of αDiar

A study was conducted to validate whether the searched results of OPs could successfully and automatically initialize the auto-planning module. It may also validate whether the fully automated treatment plans could meet clinical criteria. From April 17 to July 16, 2020, 96 lung cancer patients treated in our department were selected and analyzed. The prescription dose of all the treatment plannings was 60 Gy.

αDiar was used on each of the 96 cases and three most similar plans (denoted as Search 1, 2 and 3) were retrieved. The OPs of the three plans were respectively and manually fed into the auto-planning module in Pinnacle TPS. Three treatment plans were eventually generated for each of the 96 cases. The best one was denoted as *AP*_*AI*_. The quality of the plans was evaluated according to the criteria set forth by the Radiation Therapy Oncology Group (RTOG) [39], the National Comprehensive Cancer Network (NCCN) [40], as well as the clinical standard used in our department. For the purpose of comparison, the corresponding treatment plans which were clinically delivered to patients were also collected and denoted as *AP*_*Clinical*_.

### Comparison of treatment planning with or without αDiar

It was not likely that all *AP*_*AI*_ plans met the clinical criteria. Thus, a study was also conducted to investigate whether *AP*_*AI*_ could assist dosimetrists in clinical practice. From April 25, 2018 to March 5, 2020, 26 patients were involved in this comparison experiment. *AP*_*AI*_ plans of all patients did not meet the clinical criteria. Based on these *AP*_*AI*_ plans, two junior dosimetrists independently modified the searched OPs once and performed the plan optimization with the auto-planning module. These optimized treatment plans were denoted as $AP_{AI+\text{Human}}$. Separately, the same two dosimetrists designed the treatment plans from scratch using the same CT images, PTV and OARs. These plans were denoted as *AP*_*Human*_.

This experiment practically consisted of two phases, and they were separated by a 4-week wash-out time. In the first phase, $AP_{AI+\text{Human}}$ plans were designed for 13 of 26 cases (group A), and *AP*_*Human*_ plans were designed for the remaining 13 patients (group B). In the second phase, *AP*_*Human*_ plans were designed for group A and $AP_{AI+\text{Human}}$ plans were designed for group B. During these two phases, cases were randomly ranked across group A and group B.

### Metrics

#### NDCG

Normalized discounted cumulative gain (NDCG) was defined as

6 $$NDCG_{K}=\frac{DCG_{K}}{iDCG_{K}}$$

7 $$DCG_{K}={\sum }_{i=1}^{k}\frac{2^{r\left(l\right)}-1}{lb\left(i+1\right)}$$

where *NDCGK* was the cumulative gain of the first K positions, *lb*(*i* + 1) was the reciprocal of the impact factor of the solution at the i position, and *r*(*l*) was the relevance level of Search 1.

#### D2, D98 and D99

D2, D98 and D99 (units: Gy) were the radiation doses delivered to 2, 98 and 99% of PTV, respectively.

#### CI and HI

Conformity index (CI) [41] was defined as:

8 $$CI=\frac{V_{T{,}\mathrm{ref}}}{V_{T}}\times \frac{V_{T{,}\mathrm{ref}}}{V_{\mathrm{ref}}}$$

where *V*_*T*,*ref*_ was the volume of PTV covered by prescription dose, *V*_*T*_ was the volume of PTV, and *V*_*ref*_ was the volume covered by prescription dose.

Homogeneity index (HI) [42] was defined as:

9 $$HI=\frac{D2-D98}{D_{P}}$$

where *D*_*P*_ was the prescription dose.

#### V5, V20, V30, V40, V45 and V60

V5, V20, V30, V40, V45, and V60 were the volume percentages of OARs receiving over 5 Gy, 20 Gy, 30 Gy, 40 Gy, 45 Gy and 60 Gy, respectively.

#### MLD, MHD, Dmean and Dmax

Mean lung dose (MLD) was the mean dose of total lung, and mean heart dose (MHD) was the mean dose of heart. Dmax and Dmean were the maximum dose and mean dose of PTV or OARs, respectively.

#### Metrics’ usage

As shown in Table 1, the following metrics were used to evaluate the differences between plans. For example, D2, D98, D99 and CI and HI were used for evaluating PTV only. V5 and V20 were applied to evaluate total lung.

**Table 1:** Metrics used in different regions

Metrics	PTV	Total lung	Spinal cord	Heart
D2%	✓	–	–	–
D98%	✓	–	–	–
D99%	✓	–	–	–
CI	✓	–	–	–
HI	✓	–	–	–
V5	–	✓	–	–
V20	–	✓	–	–
MLD	–	✓	–	–
V30	–	–	–	✓
V40	–	–	–	✓
V45	–	–	–	✓
V60	–	–	–	✓
MHD	–	–	–	✓
Dmean	✓	–	–	–
Dmax	–	–	✓	–

*CI* conformity index, *HI* homogeneity index, *MHD* mean heart dose, *MLD* mean lung dose, *PTV* planning target volume

### Statistical analysis

Data analyses were performed with SPSS 20.0 (IBM Corp., Armonk, NY, USA) statistical software. For normally distributed data, paired samples t‑test was used to compare the differences of dosimetric parameters between two groups. Wilcoxon signed rank test was used to compare the differences of dosimetric parameters between two groups for the data with nonnormal distribution. *p* < 0.05 was considered statistically significant.

## Results

### Validation of the search model

In order to validate the performance of the proposed searching model, NDCG was employed. It was designed for ranking tasks with more than one relevance level. NDCG ranged from 0 to 1. The closer NDCG is to 1, the higher the accuracy is. In this research, the NDCG of the first three search results was 0.69 ± 0.09.

### Experiment of the automated usage of αDiar

#### Three-dimensional dose distribution

**Fig. 4:**
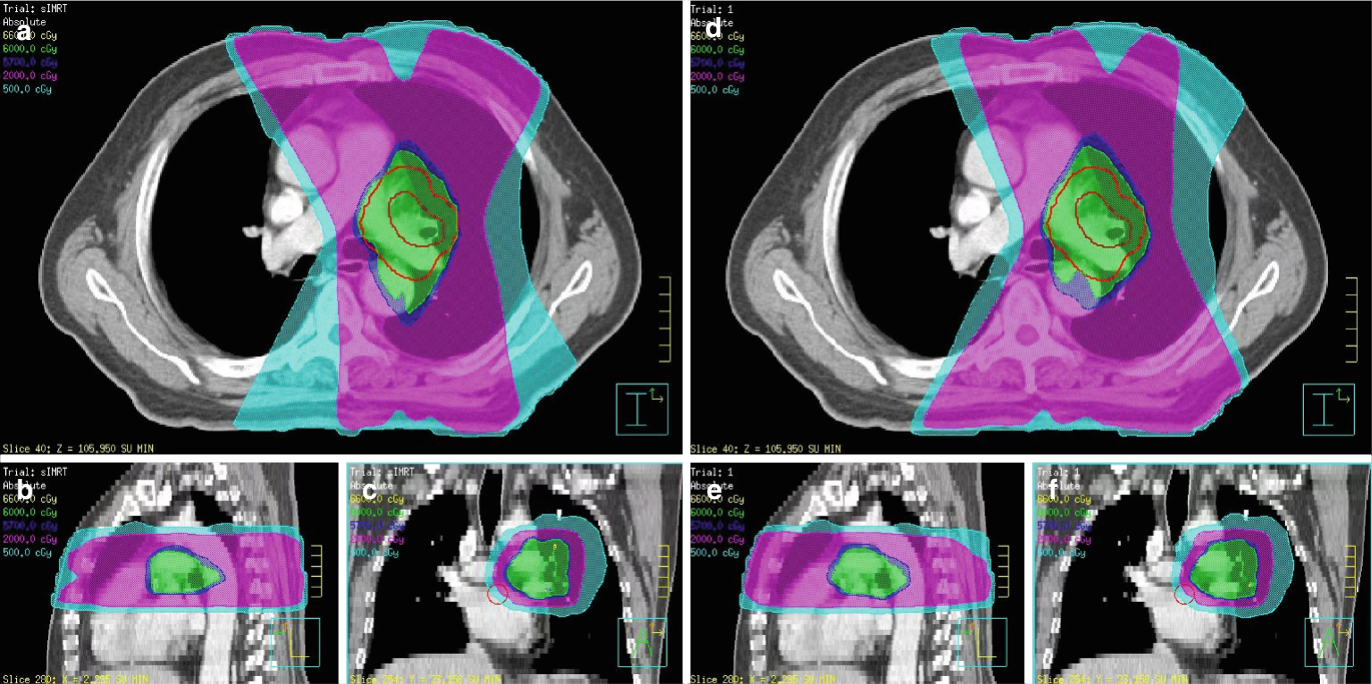
Dose distribution of AP_Clinical_ (**a**, **b**, **c**) and AP_AI_ (**d**, **e**, **f**) in the same location for one patient

**Fig. 5:**
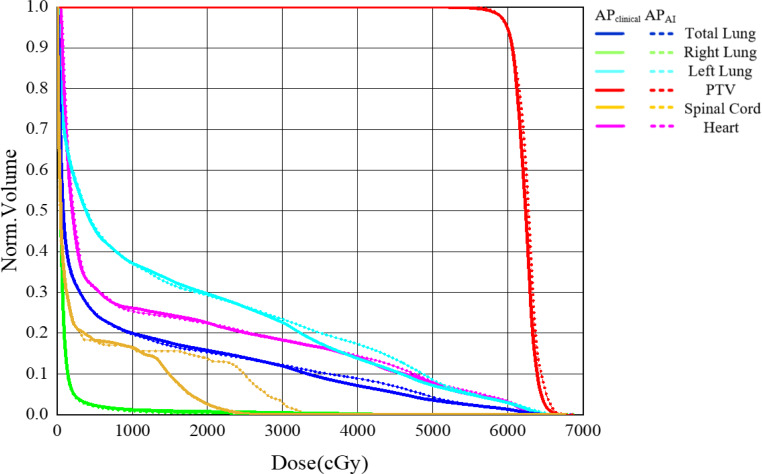
Dose–volume histogram of AP_Clinical_ (*solid* *line*) and AP_AI_ (*dotted* *line*) for the same patient as in Fig. 4. *PTV* planning target volume

**Fig. 6:**
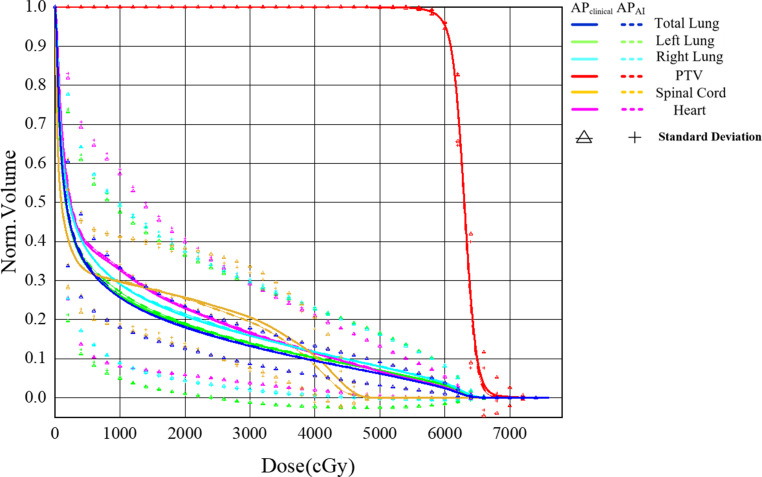
The mean dose–volume histogram (DVH) of 96 patients. *PTV* planning target volume

In Fig. 4 the dose distribution of a randomly selected patient from the 96 patients is shown, where Fig. 4a, b, c represent the dose distribution of *AP*_*Clinical*_, while Fig. 4d, e, f represent the dose distribution of *AP*_*AI*_. Figure 5 shows the difference of DVH between *AP*_*Clinical*_ and *AP*_*AI*_ for the same patient as in Fig. 4. The difference between *AP*_*Clinical*_ and *AP*_*AI*_ was small for PTV and OARs except for spinal cord. To further compare the difference in DVH between *AP*_*Clinical*_ and *AP*_*AI*_, we calculated the DVH of all patients and plotted a mean DVH (Fig. 6).

#### Comparison of dosimetric parameters between AP_Clinical_ and AP_AI_

**Fig. 7:**
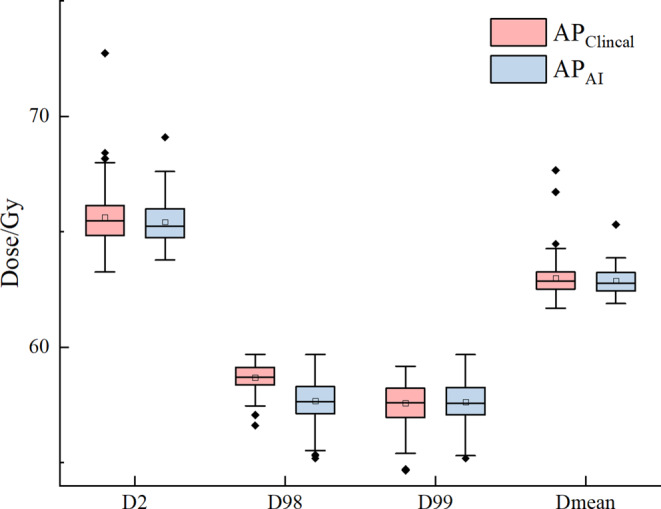
Difference of D2, D98, D99 and Dmean for PTV between AP_Clinical_ and AP_AI_

Table 2 shows the comparison results of PTV in *AP*_*Clinical*_ and *AP*_*AI*_ plans for the 96 clinical cases. D2, D98 and Dmean in *AP*_*Clinical*_ were slightly higher than those in *AP*_*AI*_ plans. There were no significant differences except D98 and HI. Figure 7 shows the difference of D2, D98, D99 and Dmean between *AP*_*Clinical*_ and *AP*_*AI*_. Overall, the evaluation metrics of *AP*_*Clinical*_ and *AP*_*AI*_ plans were similar.

**Table 2:** PTV metrics of AP_Clinical_ and AP_AI_ plans of 96 cases, and their organs at risk (OAR) metrics comparing to three standards (RTOG0623, NCCN, the standard in our department)

	AP_Clinical_	AP_AI_	*p* value
*PTV*			
D2%	65.63 ± 1.23	65.43 ± 0.96	0.31
D98%	58.69 ± 0.58	57.67 ± 0.95	*<* *0.001*
D99%	57.57 ± 0.92	57.62 ± 0.93	0.57
D_mean_	63.00 ± 0.82	62.87 ± 0.57	0.51
CI	0.71 ± 0.07	0.69 ± 0.10	0.10
HI	0.12 ± 0.03	0.13 ± 0.03	*<* *0.001*

*CI* conformity index, *HI* homogeneity index, *NCCN* national comprehensive cancer network, *PTV* planning target volume, *RTOG* Radiation Therapy Oncology Group

**Fig. 8:**
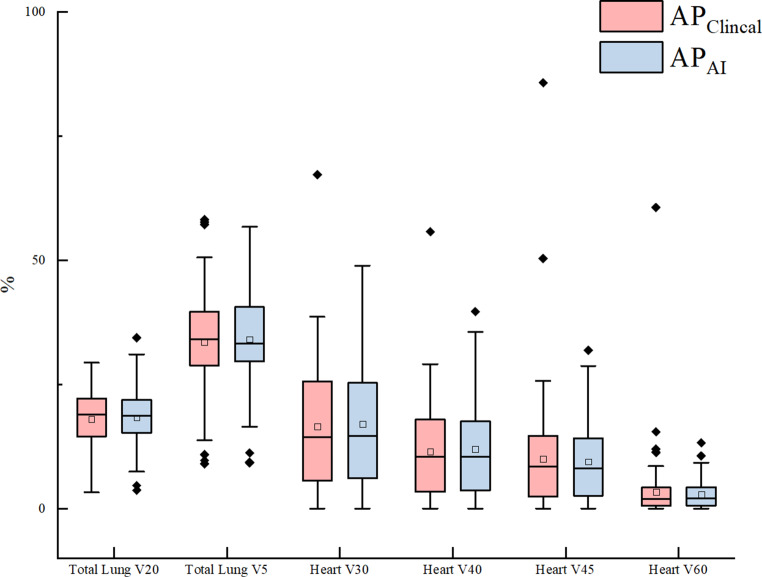
Difference of V20, V5 of total lung and V30, V40, V45, V60 of heart between AP_Clinical_ and AP_AI_

Figure 8 shows the difference of V20, V5 of total lung and V30, V40, V45, V60 of heart between *AP*_*Clinical*_ and *AP*_*AI*_. As shown in Table 3, the metrics in *AP*_*AI*_ and *AP*_*Clinical*_ of all patients met the criteria of RTOG0623 [39], except for Dmax of spinal cord. 52 *AP*_*AI*_ plans and 40 *AP*_*Clinical*_ plans met the dosimetric criteria of RTOG0623 [39]. Among the 44 *AP*_*AI*_ plans which did not meet the spinal cord dosimetric criterion in RTOG0623 [39], 33 of the corresponding *AP*_*Clinical*_ plans did also not meet the spinal cord criterion. All metrics of 92 *AP*_*Clinical*_ plans and 89 *AP*_*AI*_ plans met the dosimetric criteria set in NCCN [40]. More *AP*_*Clinical*_ plans met the clinical dosimetric criteria followed in our department, except for spinal cord (40 vs 52).

**Table 3:** OARs metrics of AP_Clinical_ and AP_AI_ plans in 96 cases, and comparison with three standards (RTOG0623, NCCN, the standard in our department)

	Criteria	AP_Clinical_^a^	AP_AI_^a^	AP_Clinical_	AP_AI_	*p* value
*RTOG0623*						
Spinal lord	Dmax ≤ 45 Gy	40	52	43.02 ± 8.78	41.79 ± 8.92	*0.02*
Total lung	V20 < 37%	96	96	17.98 ± 5.49	18.29 ± 5.61	0.62
Total lung	MLD < 20 Gy	96	96	10.05 ± 2.95	10.20 ± 3.01	*0.04*
Heart	V40 < 100%	96	96	11.47 ± 9.48	11.90 ± 9.83	0.50
Heart	V45 < 67%	96	96	9.95 ± 11.18	9.41 ± 7.84	0.74
Heart	V60 < 33%	96	96	3.36 ± 6.63	2.80 ± 2.78	0.76
*NCCN*						
Spinal cord	Dmax ≤ 50 Gy	96	94	43.02 ± 8.78	41.79 ± 8.92	*0.02*
Total lung	V20 < 35%	96	96	17.98 ± 5.49	18.29 ± 5.61	0.62
Total lung	V5 < 65%	96	96	33.55 ± 9.89	34.06 ± 9.78	0.53
Total lung	MLD ≤ 20 Gy	96	96	10.05 ± 2.95	10.20 ± 3.01	*0.04*
Heart	V40 < 80%	96	96	11.47 ± 9.48	11.90 ± 9.83	0.50
Heart	V45 < 60%	96	96	9.95 ± 11.18	9.41 ± 7.84	0.74
Heart	V60 < 30%	96	96	3.36 ± 6.63	2.80 ± 2.78	0.76
Heart	MHD ≤ 26 Gy	92	91	12.12 ± 8.12	12.73 ± 9.86	*0.04*
*Our department*						
Spinal cord	Dmax ≤ 45 Gy	40	52	43.02 ± 8.78	41.79 ± 8.92	*0.02*
Total lung	V20 < 25%	90	85	17.98 ± 5.49	18.29 ± 5.61	0.62
Total lung	V5 < 45%	86	83	33.55 ± 9.89	34.06 ± 9.78	0.53
Total lung	MLD ≤ 15 Gy	94	93	10.05 ± 2.95	10.20 ± 3.01	*0.04*
Heart	V30 < 40%	95	89	16.53 ± 12.74	16.95 ± 13.31	0.62
Heart	V40 < 30%	95	90	11.47 ± 9.48	11.90 ± 9.83	0.50
Heart	MHD ≤ 26 Gy	92	91	12.12 ± 8.12	12.73 ± 9.86	–

*MHD* mean heart dose, *MLD* mean lung dose, *NCCN* national comprehensive cancer network, *OAR* organs at risk, *RTOG* Radiation Therapy Oncology Group

^a^Number of the individual plans achieving the standards

**Fig. 9:**
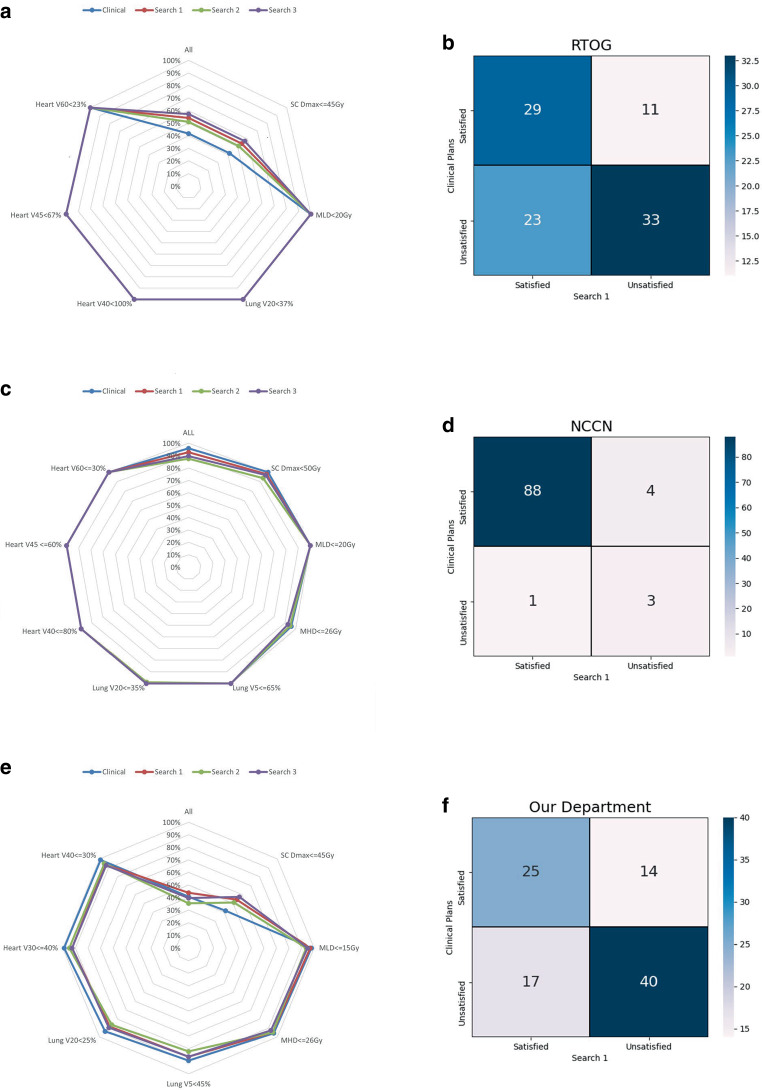
The percentage of plans met RTOG0623 (RTOG: Radiation Therapy Oncology Group;** a**), National Comprehensive Cancer Network (NCCN; **c**), and the standard in our department (**e**). Confusion matrix of clinical plans versus Search 1 (the plan generated from the most similar optimization parameters) based on RTOG0623 (**b**), NCCN (**d**), and the standard in our department (**f**)

As shown in Fig. 9a, c and e, the percentages of *AP*_*AI*_ and *AP*_*Clinical*_ plans that met all of the RTOG0623 [39] dosimetric criteria were 54.17 and 41.67%, respectively. The percentage of *AP*_*Clinical*_ plans (95.83%) meeting the NCCN criteria [40] was slightly higher than that of *AP*_*AI*_ (92.71%). The percentages of *AP*_*AI*_ and *AP*_*Clinical*_ plans that met the dosimetric criteria of our department were 43.75 and 40.63%, respectively. The figures also showed that the numbers of plans meeting the criteria were similar between *AP*_*AI*_ and *AP*_*Clinical*_. Slightly more *AP*_*AI*_ plans met the criteria of RTOG0623 [39] and our department standards than the *AP*_*Clinical*_ plans, but slightly fewer *AP*_*AI*_ than the *AP*_*Clinical*_ plans that met the NCCN criteria [40].

As shown in Fig. 9b, d and f, the numbers of *AP*_*AI*_ plans that met the dosimetric criteria of RTOG0623[39], NCCN [40] and our department standards were 52, 89 and 42, respectively. The number of cases in which both *AP*_*AI*_ and *AP*_*Clinical*_ met the three criteria was 29, 88 and 25, respectively. The number of cases that *AP*_*AI*_ plans met the RTOG0623 [39] criteria while the corresponding *AP*_*Clinical*_ plans did not meet the criteria was 23. This was higher than the number of cases of *AP*_*Clinical*_ plans that met RTOG0623 [39] criteria but the corresponding *AP*_*AI*_ plans did not. Similar results were obtained when the dosimetric criteria of our department were adopted. For the NCCN [40] dosimetric criteria, the number of cases that *AP*_*AI*_ met the criteria but *AP*_*Clinical*_ did not was fewer than the number of cases that *AP*_*Clinical*_ met the criteria but *AP*_*AI*_ did not. However, these two numbers were very close.

An experienced radiation oncologist reviewed all *AP*_*AI*_ and *AP*_*Clinical*_ plans and was blinded to the information about how these plans were designed. Based on this evaluation, 9 *AP*_*AI*_ plans were better than *AP*_*Clinical*_, 43 *AP*_*AI*_ plans were similar to *AP*_*Clinical*_, and 44 were worse. In conclusion, 54.17% of the *AP*_*AI*_ were better than or comparable to the *AP*_*Clinical*_, and could be directly applied in clinical practice.

### Comparison experiment

#### Three-dimensional dose distribution

**Fig. 10:**
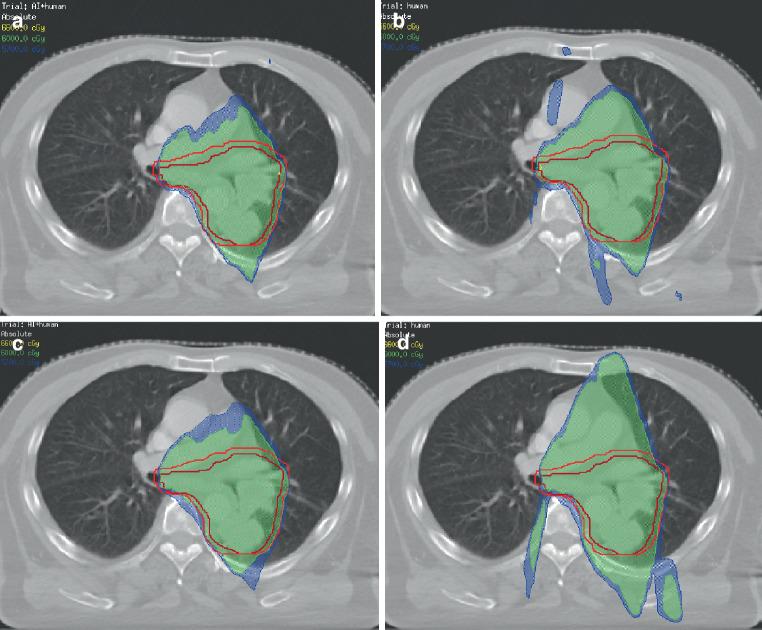
Example of isodose distribution for one patient: **a** AP_AI_ _+_ _Human_ designed by dosimetrist A, **b** AP_Human_ designed by dosimetrist A, **c** AP_AI_ _+_ _Human_ designed by dosimetrist B, **d** AP_Human_ designed by dosimetrist B

The dose distribution of a randomly selected patient from the 26 patients is shown in Fig. 10, (a) the dose distribution of $AP_{AI+\text{Human}}$ designed by dosimetrist A, (b) the dose distribution of *AP*_*Human*_ designed by dosimetrist A, (c) the dose distribution of $AP_{AI+\text{Human}}$ designed by dosimetrist B, and (d) the dose distribution of *AP*_*Human*_ designed by dosimetrist B. Visually, for dosimetrist A and B, the dose distribution of $AP_{AI+\text{Human}}$ was significantly better than *AP*_*Human*_.

#### Comparison of dosimetric parameters between $\boldsymbol{AP}_{\boldsymbol{AI}+\textbf{Human}}$ and AP_Human_

Table 4 shows the metrics of the $AP_{AI+\text{Human}}$ and *AP*_*Human*_ plans. All metrics of PTV in $AP_{AI+\text{Human}}$ plans designed by dosimetrist A were better than those of *AP*_*Human*_. For dosimetrist B, all PTV metrics of $AP_{AI+\text{Human}}$ plans were better than those of *AP*_*Human*_ , except HI.

**Table 4:** Metrics results of planning target volume (PTV) and organs at risk (OARs) in AP_AI_ _+_ _Human_ and AP_Human_

	Dosimetrist A			Dosimetrist B		
	AP_AI_ _+_ _Human_	AP_Human_	*p* value	AP_AI_ _+_ _Human_	AP_Human_	*p* value
*PTV*						
D2%	65.30 ± 1.47	65.76 ± 1.69	*0.02*	65.30 ± 1.48	65.42 ± 0.77	0.05
D98%	58.72 ± 0.44	58.68 ± 0.80	0.82	58.72 ± 0.61	58.64 ± 0.64	0.68
D99%	57.58 ± 0.74	57.42 ± 1.74	0.80	57.55 ± 0.97	57.42 ± 0.96	0.68
Dmean	62.73 ± 0.42	63.05 ± 0.94	*0.04*	61.33 ± 7.67	62.95 ± 0.45	*0.01*
CI	0.67 ± 0.09	0.63 ± 0.11	*0.04*	0.67 ± 0.10	0.62 ± 0.10	*0.01*
HI	0.11 ± 0.03	0.12 ± 0.04	0.18	0.11 ± 0.03	0.11 ± 0.02	0.08
*Total lung*						
V5	40.89 ± 10.57	41.10 ± 9.46	0.83	41.96 ± 11.47	38.34 ± 9.11	*0.01*
V20	22.34 ± 4.41	22.89 ± 4.40	0.12	22.34 ± 4.44	22.29 ± 4.49	0.86
MLD	12.54 ± 2.50	12.71 ± 2.50	0.16	12.48 ± 2.49	14.31 ± 10.63	0.16
*Heart*						
V30	22.94 ± 14.43	22.07 ± 13.69	0.34	22.84 ± 14.38	21.88 ± 12.51	0.70
V40	17.59 ± 12.31	16.92 ± 11.64	0.17	17.54 ± 11.89	16.80 ± 10.16	0.97
V45	14.14 ± 10.38	14.27 ± 10.07	0.93	14.09 ± 9.97	14.39 ± 8.89	0.18
V60	3.82 ± 3.19	5.03 ± 4.13	*<* *0.001*	3.69 ± 2.96	5.11 ± 4.02	*0.01*
MHD	15.71 ± 8.23	15.46 ± 8.48	0.49	21.12 ± 12.33	20.74 ± 12.28	0.81
*Spinal cord*						
Dmax	44.74 ± 5.15	44.87 ± 5.45	0.80	46.33 ± 5.45	46.74 ± 4.84	0.13

*CI* conformity index, *HI* homogeneity index, *MHD* mean heart dose, *MLD* mean lung dose, *PTV* planning target volume

As shown in Table 4, the metrics of total lung and spinal cord in $AP_{AI+\text{Human}}$ plans designed by dosimetrist A were slightly better than those in *AP*_*Human*_. For the plans designed by dosimetrist A, V45 and V60 of heart in $AP_{AI+\text{Human}}$ were slightly better than those in the corresponding *AP*_*Human*_. V30, V40 and MHD of heart in $AP_{AI+\text{Human}}$ were slightly worse than those in the corresponding *AP*_*Human*_. MLD of total lung, V45 and V60 of heart and Dmax of spinal cord in $AP_{AI+\text{Human}}$ designed by dosimetrist B were better than those in the corresponding *AP*_*Human*_.

## Discussion

In this study, a novel architecture to automatically retrieve treatment plans in the database via the agent of virtual dose images was proposed. As a knowledge-based method to implement an automated design of planning for lung cancer patients treated with IMRT, the virtual dose images were inferred from the masks of PTV and OARs. And the whole procedure of retrieval and planning can be implemented in a fully automated system. In order to validate the performance of αDiar, two experiments were conducted. The first experiment was to investigate the quality of αDiar-initialized plans without any planner intervention, and the second experiment was to compare the impact on the planning quality with and without the aid of αDiar. The first experiment revealed that over half of the tested αDiar-initialized plans could be directly used in clinical practice, and the second experiment revealed that the αDiar-initialized planning procedure could improve plan qualities.

**Fig. 11:**
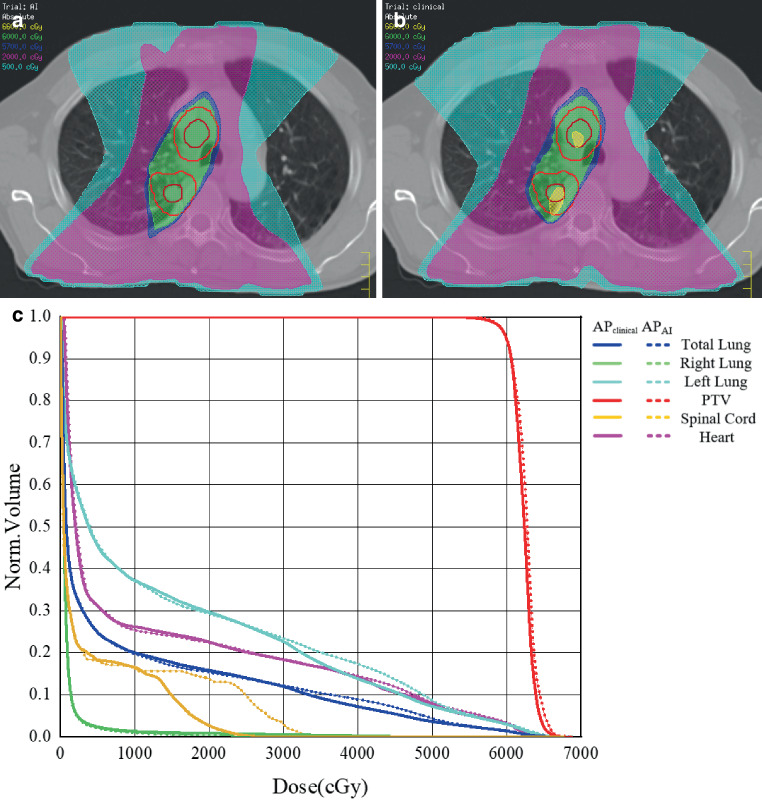
Isodose distribution and dose–volume histogram (DVH) of one patient’s AP_AI_ and AP_Clinical_: **a** isodose distribution of AP_AI_; **b** isodose distribution of AP_Clinical_; **c** DVH comparison between AP_AI_ (*dashed* *lines*) and AP_Clinical_ (*solid* *lines*). *PTV* planning target volume

A comparison of isodose distributions and DVH of *AP*_*AI*_ and *AP*_*Clinical*_ plans for one patient are shown in Fig. 11. The results showed that although *AP*_*AI*_ and *AP*_*Clinical*_ plans met the clinical requirements, quality differences still existed. And the use of αDiar may lead to better quality.

In 44 cases (PTV size: 251.93 ± 117.48cc), the qualities of *AP*_*AI*_ plans were worse than those of the corresponding *AP*_*Clinical*_ plans. Compared with *AP*_*Clinical*_ plan, 13 *AP*_*AI*_ plans exhibited worse conformability despite the lower doses received by OARs. In one *AP*_*AI*_ plan, the metrics of the plan met all dosimetric criteria but failed to provide individualized protection to the unilateral lung. The doses of OARs in 30 *AP*_*AI*_ plans were higher than those in the corresponding *AP*_*Clinical*_ plans. It was observed that a larger PTV often led to poorer quality of *AP*_*AI*_ plans than their corresponding *AP*_*Clinical*_ plans. The *AP*_*AI*_ plans tended to over-protect OARs, while decreasing the conformability. Moreover, the αDiar process could not consider the oncologists’ preferences which should be pursued in the future research.

In this study, one case (PTV size: 119.70cc) was excluded because its OPs could not be used in auto-planning module. Since the OPs did not present any abnormality, this may be due to an internal error in Pinnacle, which needs further investigation.

In the second experiment, two junior dosimetrists designed plans for 26 lung cancer patients with and without the assistance of αDiar, respectively. Each plan was optimized only once. For dosimetrist B, based on the results of metrics, the qualities of treatment plans [43] designed without αDiar were generally inferior to those designed by dosimetrist A. However, the quality differences of the plans initiated with αDiar decreased remarkably between the two dosimetrists, which showed that the αDiar process may have the potential to improve quality differences between planners. Figure 10a and b displays the isodose distributions of a case designed by dosimetrist A with and without αDiar. Figure 10c and d displays the corresponding isodose distributions designed by dosimetrist B. Evidently, isodose distributions were improved with the use of αDiar.

As an image retrieval architecture, αDiar could be very useful in taking advantage of the entire treatment plans database in the department of radiation oncology and making it available as a knowledge base which can be accessed by all dosimetrists in the future. This architecture may not only make it possible to “share” the knowledge of experienced dosimetrists, but also help to improve the overall qualities of treatment plans.

The utilization of αDiar may change the workflow of radiotherapy treatment planning. In the current workflow, upon the radiation oncologist determined the prescription and approved the contours of PTV and OAR, the dosimetrist designed the treatment plan on TPS by configuring the prescription, dose calculation algorithm and grid resolution, optimization algorithm and OPs. The treatment planning design process was iterated until the treatment plan was clinically acceptable. Upon implementation of αDiar, the workflow may be changed as follows. If the retrieved OPs could be used to generate a satisfactory treatment plan, the plan could be directly applied to clinical treatment with the approval of the dosimetrist and radiation oncologist. If the αDiar-initialized plan does not meet the clinical requirements, it could also help dosimetrists to start with a semi-ready plan to achieve a plan that meets clinical standards.

This proposed architecture provides a scenario where no patients’ images are transferred out of hospitals. In the process, a patient’s CT scan images are transferred to the in-hospital workstation for the purpose of rigid registration, and the transformation matrix gained from the registration is employed to transform the masks of PTV and OARs. The registered masks of PTV and OARs are utilized to predict virtual dose images which serve as substitutes for CT scans in content-based image retrieval (CBIR). Once the most similar clinical dose images are found in the database, a link between the searching plan and the stored plan in the database is established, and the corresponding stored OPs can be transferred and applied to the new plan. Furthermore, redundant information in CT scans is not necessary for image retrieval. For example, the anatomic information of muscles, bones, vessels, and airways may lead to over-complicated AI-model training. Replacing these anatomic structures with OAR masks as well as low-dose areas in dose images can simplify the training of the image retrieval model and increase the searching speed.

Traditionally, the training of a CBIR system has often been challenged by the lack of similar pairs of samples [44]. In a database with T samples, a physician theoretically needed to review T pairs of samples to find the most similar one. Finding exhaustive pairs of possibly similar samples in a database with T samples required T×T times of review, which was time-consuming and labor-intensive. As proposed in this research, the virtual dose images and their clinical dose images were naturally a similar pair. Thus, the labor-intensive work of identifying similar pairs to train the CBIR model could be avoided by utilizing virtual dose images as the agent.

In the future, the manual input of OPs to the Pinnacle user interface could be replaced by engineering work to embed αDiar in a TPS. Also, due to the knowledge-based method, the performance of αDiar can be expected to improve by expanding the database size without changing the AI models. Compared with the commercial KBP method, the database of αDiar can be expanded to a larger volume. On the other hand, the robustness and feasibility of αDiar still needed further improvement as well as generality through the implementation of αDiar in other institutions. Finally, in this study, there were two layers of auto-planning. One was the proposed in-house KBAP that produced OPs, and the other was the commercial auto-planning module in Pinnacle. At present, we cannot decouple the effect of the first from the second. However, due to the extensive validation of the two layers, the results were credible when comparing the dosimetric parameters.

## Conclusion

In this article, the authors proposed a novel knowledge-based architecture for an automated treatment plan design named αDiar. It can automatically retrieve radiotherapy treatment plans from the database through proxy virtual dose images. It was found that 54% of lung cancer patients can be treated with radiotherapy treatment plans that were generated using the fully automated αDiar. The plan quality and interplanner plan quality variation can also be improved with the architecture. The implementation of αDiar may change the radiotherapy workflow. Further investigation is required.
